# Case Report: Integration of intratumoral oncolytic adenovirus with total neoadjuvant therapy in metachronous locally advanced rectal cancer

**DOI:** 10.3389/fonc.2026.1765097

**Published:** 2026-04-07

**Authors:** Xiufeng Chen, Zhou Zhao, Zhixiong Chen, Wei Li, Hao Sun

**Affiliations:** 1 Gastrointestinal Cancer Center, Chongqing University Cancer Hospital, Chongqing, China; 2 Chongqing Key Laboratory for the Mechanism and Intervention of Cancer Metastasis, Chongqing University Cancer Hospital, Chongqing, China

**Keywords:** oncolytic virotherapy, precision oncology, rectal cancer, sphincter preserving, total neoadjuvant therapy

## Abstract

**Background:**

Recent landmark randomized controlled trials have established total neoadjuvant therapy (TNT) as a standard treatment option for selected patients with locally advanced rectal cancer (LARC), offering improved tumor response and opportunities for organ preservation. In parallel, the integration of oncolytic virotherapy (OVT) into neoadjuvant regimens has shown potential benefits in various solid tumors. However, to date, no clinical reports have demonstrated the therapeutic efficacy of combining OVT with TNT specifically in LARC.

**Case presentation:**

We report a case of a 50-year-old patient diagnosed with metachronous LARC harboring TP53 mutation. We treated this patient with intratumoral injection of the recombinant human adenovirus H101 in combination with TNT, resulting in a pathologic complete response (pCR) and successful preservation of anal sphincter function. At the most recent follow-up, there was no evidence of local recurrence or distant metastasis.

**Conclusion:**

This case highlights the potential of oncolytic virotherapy to enhance chemotherapy and radiotherapy effects in LARC patients.

## Introduction

Colorectal cancer (CRC) is the third most common malignancy and the second leading cause of cancer-related mortality worldwide (1). Rectal cancer accounts for approximately 30% of all CRC cases, with LARC posing significant therapeutic challenges (2). The current standard of treatment typically involves neoadjuvant chemoradiotherapy (nCRT) followed by total mesorectal excision and adjuvant chemotherapy. However, recent advances in total neoadjuvant therapy (TNT)—which integrates both chemotherapy and radiotherapy in the preoperative phase—have improved rates of tumor response and organ-preserving in selected patients (3–5). Current studies report pathological complete response (pCR), defined as the absence of residual tumor on histopathological examination, in up to 33% of patients treated with TNT (3–6). In addition, patients who achieve a clinical complete response (cCR), characterized by no detectable tumor on imaging and endoscopic assessment, may be managed with a non-operative “watch-and-wait” (W&W) strategy with oncologic outcomes comparable to surgery. W&W strategy minimize treatment-related morbidity and preserving anorectal function (7, 8). Obviously, these findings have opened new avenues for function-preserving treatment paradigms in highly selected LARC cases. Oncolytic virotherapy (OVT) represents a novel and promising therapeutic modality in oncology, harnessing viruses’ natural or engineered ability to selectively infect and destroy tumor cells (9). Beyond direct oncolysis, oncolytic viruses modulate the tumor microenvironment by releasing tumor-associated antigens, which stimulate innate and adaptive anti-tumor immune responses (10). Generally, OVT can be administered as monotherapy or in combination with other modalities to enhance overall therapeutic efficacy (11, 12). Recombinant human adenovirus type 5 (H101, Oncorine) is the first conditionally replicating oncolytic virus approved for clinical use in China. It selectively replicates in tumor cells with defects in the p53 pathway, leading to targeted cell lysis and immunogenic tumor cell death (13–15). Especially, H101 has demonstrated synergy with standard treatments including chemotherapy and radiotherapy, making it a promising candidate for combinatorial regimens in solid tumors (11, 12, 16). However, limited studies have investigated the combination of oncolytic virotherapy with neoadjuvant therapy.

Herein, we report a case of a 50-year-old male with TP53-mutant LARC who achieved a cCR and pCR following intratumoral injection of H101 combined with TNT (five cycles of CapeOX chemotherapy and long-course chemoradiotherapy). We detail the patient’s clinical course, radiologic and pathologic responses, and short-term follow-up, and discuss the potential of combining H101 with TNT as a novel therapeutic strategy for rectal cancer with an emphasis on tumor downstaging and anal sphincter preservation.

## Case presentation

**Figure 1:**
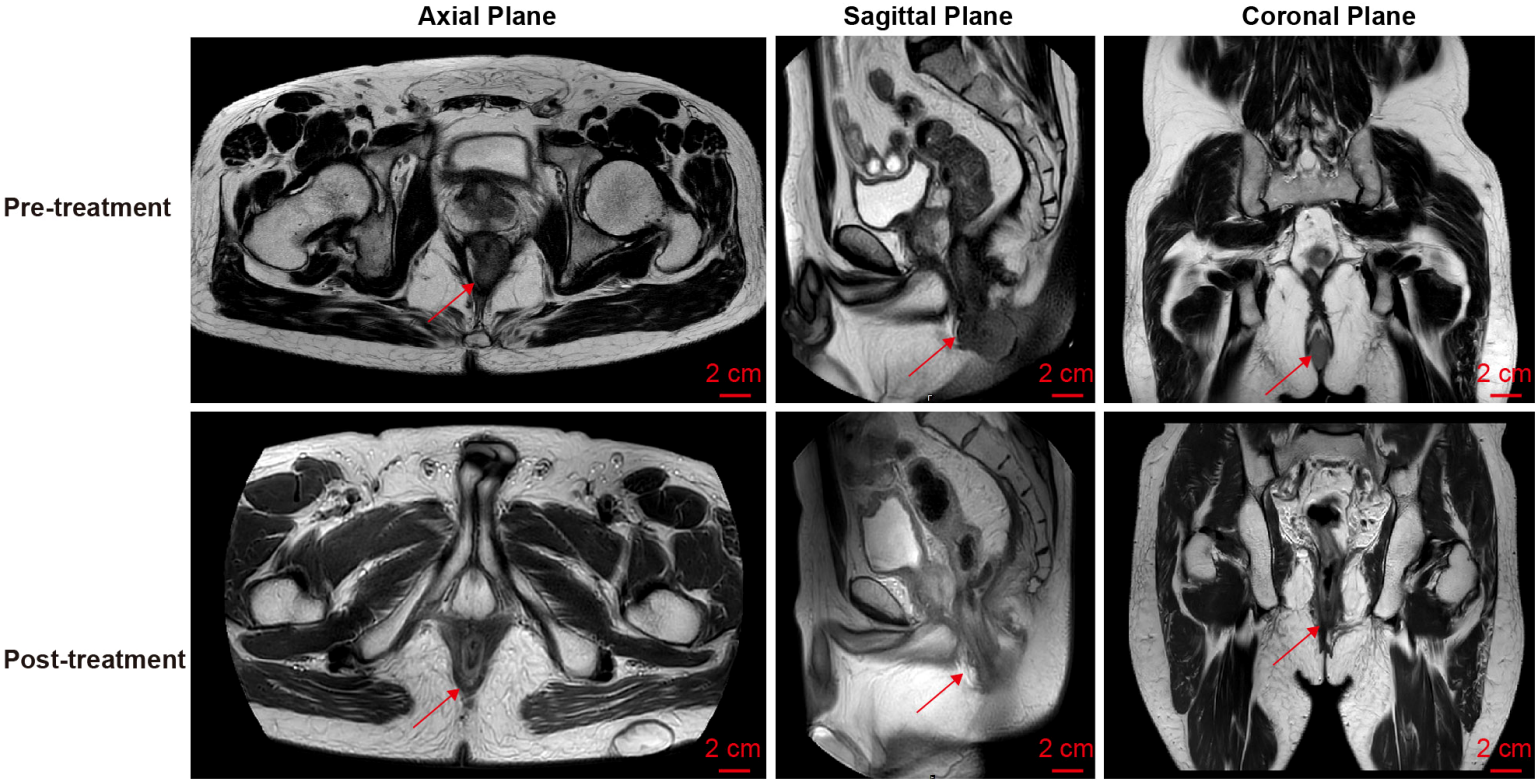
Pelvic MRI images of the metachronous rectal tumor before and after treatment. Arrows indicate the anal canal tumor before treatment (upper row) and after completion of total neoadjuvant therapy combined with intratumoral H101 (lower row).

A 50-year-old man presented on March 29, 2023, with rectal bleeding and a palpable anal mass, detected during routine follow-up one year after surgery for synchronous rectal and colonic cancers. He had no other significant symptoms. Pelvic MRI showed irregular thickening of the rectal wall and anal canal, with a lesion measuring 2.5 × 4.5 cm and no regional lymph node involvement (**Figure 1**). Clinical staging was cT3N0M0.

Colonoscopy confirmed a mass at the anal canal, and biopsy revealed rectal adenocarcinoma (**Table 1**). Immunohistochemistry (IHC) showed HER-2 (1+) and pMMR status. Genetic testing revealed microsatellite stability (MSS) status, KRAS mutation (A146V, frequency 11.14%), TP53 mutation (G245S, frequency 7.64%), and APC mutation (G129*, frequency 11.34%) (**Table 2**, **the row of “Apr 2023”**).

**Table 1:** Timeline of diagnosis and treatment.

Benign or malignant lesions at different anatomical locations	Pre-treatment		Treatment (Time)	Post-treatment	
	Endoscopy (Time)	HE stain		HE stain	Endoscopy (Time)
Mid-rectal cancer	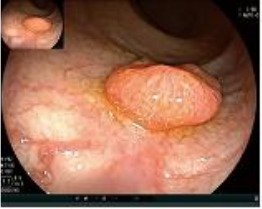 Suspicious for malignancy (2022-01-06)	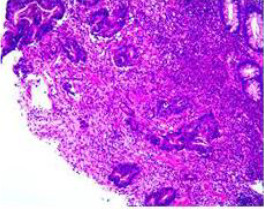 Rectal adenocarcinoma	Low anterior resection (2022-01-15)	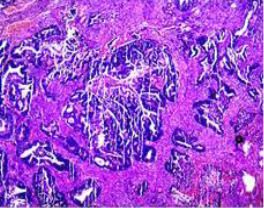 Rectal adenocarcinoma	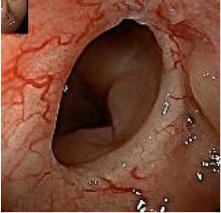 Anastomosis: healed, no recurrence (2025-09-25)
Hepatic flexure colon cancer	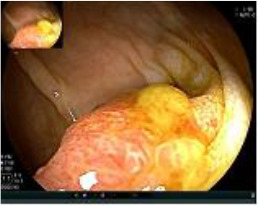 Suspicious for malignancy (2022-01-06)	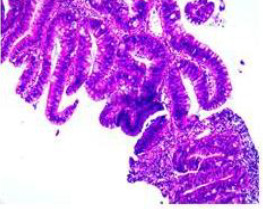 Biopsy: Villous adenoma	Re-biopsy		
	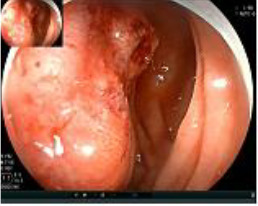 Suspicious for malignancy (2022-04-21)	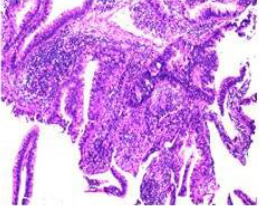 Re-biopsy: colonic adenocarcinoma	Right hemicolectomy (CME) (2022-04-24)	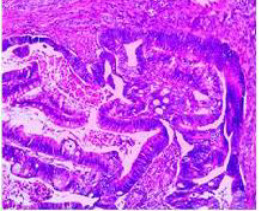 Pathology: Colonic adenocarcinoma	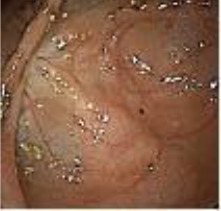 Anastomosis: healed, no recurrence (2025-09-25)
Benign colonic lesion	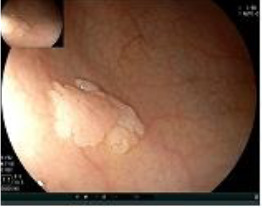 Sigmoid lesion (2022-01-06)		Endoscopic resection (2022-04-21)	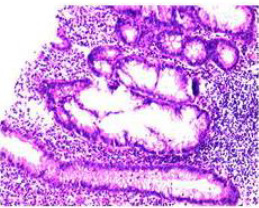 Tubular adenoma	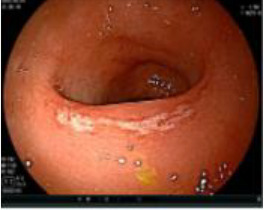 Well-healed surgical site (2022-09-13)
	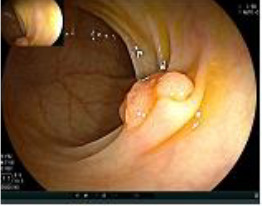 Descending colon lesion (2022-01-06)		Endoscopic resection (2022-04-21)	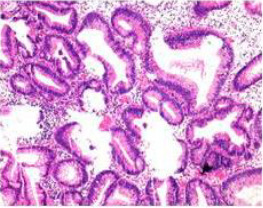 Pathology: Tubular adenoma	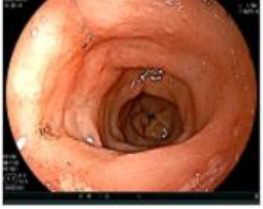 Well-healed surgical site (2022-09-13)
	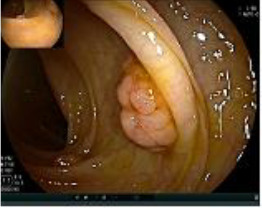 Transverse colon lesion (2022-01-06)		Endoscopic resection (2022-04-21)	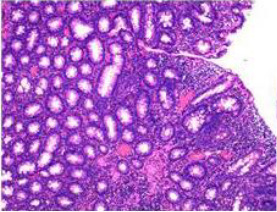 Tubular adenoma	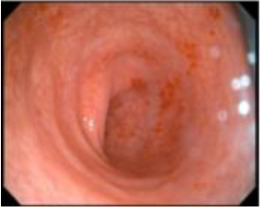 Well-healed surgical site (2022-09-13)
Anus cancer	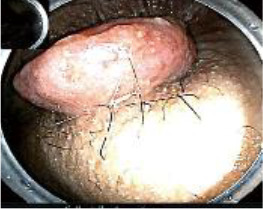 Metachronous LARC (2023-04-21)	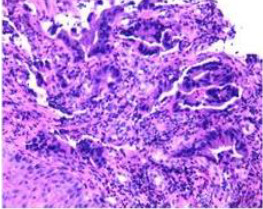 Biopsy: Rectal adenocarcinoma	OVT + TNT (Apr. 2023 to Oct. 2023); Local excision (2023-12-24)	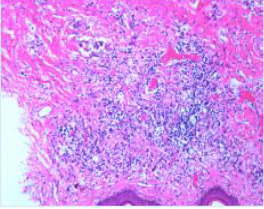 Pathology: No residual tumor; granulomatous inflammation. (2023-12-31)	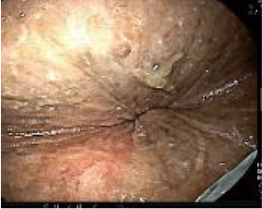 Metachronous LARC after OVT + TNT (2023-10-26)

CME, complete mesocolic excision; LARC, locally advanced rectal cancer; OVT, oncolytic virotherapy; TNT, total neoadjuvant therapy.

**Table 2:** Molecular profiling results of surgically resected tumor specimens.

Time	Site	Methods	Test items	Results
Jan 2022	Mid-rectal cancer	IHC	HER-2	1+
			MMR proteins	pMMR
		PCR + capillary electrophoresis	MSI status	MSS
		Fluorescence quantitative PCR	KRAS	K117N, A146T/V/P
			BRAF	negative
			NRAS	negative
			PIK3A	negative
May 2022	Hepatic flexure colon cancer	IHC	HER-2	negative
			MMR proteins	pMMR
		PCR + capillary electrophoresis	MSI status	MSS
		Fluorescence quantitative PCR	KRAS	G12S/D
			BRAF	negative
			NRAS	negative
			PIK3A	negative
Apr 2023	Anus cancer	Next-generation sequencing (A panel of 41 genes and MSI status)	MSI status	MSS
			KRAS	A146V
			TP53	G245S
			APC	G129*

G129*, a nonsense (stop-gain) mutation most commonly described in tumor suppressor genes APC; * = a premature stop codon; IHC, immunohistochemistry; MMR, mismatch repair; pMMR: proficient mismatch repair; dMMR: deficient mismatch repair; MSI, microsatellite instability; PCR, polymerase chain reaction.

### Past medical history

In January 2022, colonoscopy revealed a rectal lesion, a hepatic flexure mass, and multiple colonic polyps involving the sigmoid, descending, and transverse colon. Biopsy demonstrated moderately differentiated rectal adenocarcinoma, a villous adenoma at the hepatic flexure (suspicious for malignancy), and tubular adenomas at multiple colonic sites.

The pelvic MRI demonstrated distal margin of rectal adenocarcinoma located approximately 6.0 cm from the anal verge and a longitudinal extent of 4.4 cm. Colonoscopy revealed a tumor located about 7 cm from the anal verge, with a maximum diameter of 1.8 cm. Based on these findings, the lesion was classified as a mid-rectal adenocarcinoma. The patient underwent low anterior resection (Dixon procedure) on January 15, 2022. Postoperative pathology confirmed rectal adenocarcinoma (pT1N0M0, stage I). Immunohistochemistry and genetic testing results are summarized in **Table 2**. At the most recent follow-up in September 2025, endoscopic evaluations showed no evidence of tumor recurrence or distant metastasis (**Table 1**).

Endoscopic resection of the sigmoid, descending, and transverse colon lesions was performed in April 2022, and pathology confirmed tubular adenomas. All resection sites healed well on follow-up endoscopy.

Given the suspicious morphology of the hepatic flexure lesion, repeat biopsy was performed in April 2022 and confirmed colonic adenocarcinoma (**Table 1**). The patient subsequently underwent right hemicolectomy with complete mesocolic excision (CME) on April 24, 2022. Final pathology revealed pT3N0M0 (stage II) colonic adenocarcinoma with HER2-negative and proficient mismatch repair (pMMR) status. Considering the presence of synchronous stage II colorectal cancer, four cycles of adjuvant CapeOX (capecitabine plus oxaliplatin) were administered. The patient was subsequently followed with regular imaging and colonoscopic surveillance. No evidence of recurrence was detected through September 2025 (**Table 1**). The timeline of diagnosis and treatment is summarized in **Table 1**.

### Physical examination

The patient had an Eastern Cooperative Oncology Group (ECOG) performance status of 1. Abdominal examination revealed well-healed surgical scars. No other abnormal findings were noted.

### Treatment

Considering the time interval after prior curative surgery, the anatomical separation from the previous anastomotic site, the absence of local recurrence during surveillance, and the differences in molecular profiles, the newly identified anus lesion was considered more consistent with a metachronous rectal cancer rather than metastatic disease. Radical surgery without sphincter preservation was recommended; however, the patient was strongly averse to permanent colostomy and refused radical resection. Considering the patient’s prior exposure to adjuvant chemotherapy, the expected efficacy of conventional neoadjuvant chemoradiotherapy may be suboptimal. Moreover, due to the MSS/pMMR status and the absence of actionable mutations, the patient was not a candidate for immunotherapy or targeted therapy. A literature review suggested that oncolytic virotherapy may sensitize tumors to chemoradiotherapy (11). Given the tumor’s accessible location, TP53 mutation, and the patient’s willingness to explore novel therapeutic options, we proposed a treatment plan involving intratumoral injection of H101 and TNT.

The patient received a multimodal treatment regimen consisting of oncolytic virotherapy, chemotherapy, and radiotherapy, administered in 21-day cycles. During Cycles 1–3, oxaliplatin (130 mg/m² on Day 1) and capecitabine (850 mg/m² twice daily on Days 1–14) were combined with intratumoral H101 (5.0 × 10¹¹ viral particles per day on Days 1–5). Concurrent chemoradiotherapy was delivered in Cycles 4–5, with capecitabine given on Days 1–14 and intensity-modulated radiotherapy administered at 1.8 Gy per fraction. Consolidation chemotherapy with oxaliplatin and capecitabine was subsequently administered in Cycles 6–7. Surgical resection was performed after an interval of 8 weeks following completion of neoadjuvant therapy (**Table 3**).

**Table 3:** Summary of the treatment regimen.

Treatment phase (21 days per cycle)	Regimen	Schedule
Cycle 1	Oxaliplatin	130 mg/m^2^, D1
	Capecitabine	850 mg/m^2^, bid, D1-14
	H101	(5.0 × 10¹¹ vp/day, intratumoral injection, D1-5
Cycle 2	Oxaliplatin	130 mg/m^2^, D1
	Capecitabine	850 mg/m^2^, bid, D1-14
	H101	(5.0 × 10¹¹ vp/day, intratumoral injection, D1-5
Cycle 3	Oxaliplatin	130 mg/m^2^, D1
	Capecitabine	850 mg/m^2^, bid, D1-14
	H101	(5.0 × 10¹¹ vp/day, intratumoral injection, D1-5
Cycle 4	Capecitabine	850 mg/m^2^, bid, D1-14
	Radiotherapy	IMRT, 1.8 Gray, D1-5, D8-12, D15-19
Cycle 5	Capecitabine	850 mg/m^2^, bid, D1-14
	Radiotherapy	IMRT, 1.8 Gray, D1-5, D8-12, D15-17
Cycle 6	Oxaliplatin	130 mg/m^2^, D1
	Capecitabine	850 mg/m^2^, bid, D1-14
Cycle 7	Oxaliplatin	130 mg/m^2^, D1
	Capecitabine	850 mg/m^2^, bid, D1-14

bid, twice a day; IMRT, intensity-modulated radiation therapy; vp, viral particles.

### Outcome and response

Upon completion of TNT, the assessments indicated a cCR response (**the lower images of****Figure 1**; **Table 1**). The sigmoidoscopy revealed only scar tissue at the previous tumor site. In November 2023, the patient proceeded with local excision to confirm pathological status. Histopathological analysis of the resected specimen showed dense fibrosis, chronic inflammatory infiltrates, and granulomatous changes, but no residual adenocarcinoma was identified (**Table 1**). Surgical margins were negative for malignancy. The patient recovered uneventfully and was discharged without postoperative complications.

### Adverse events related to OVT

The principal adverse event encountered was a transient, which occurred within 8–24 hours following the OVT, peaking at a temperature of 38.6 °C. This fever abated following physical cooling interventions.

### Follow-up

**Figure 2:**
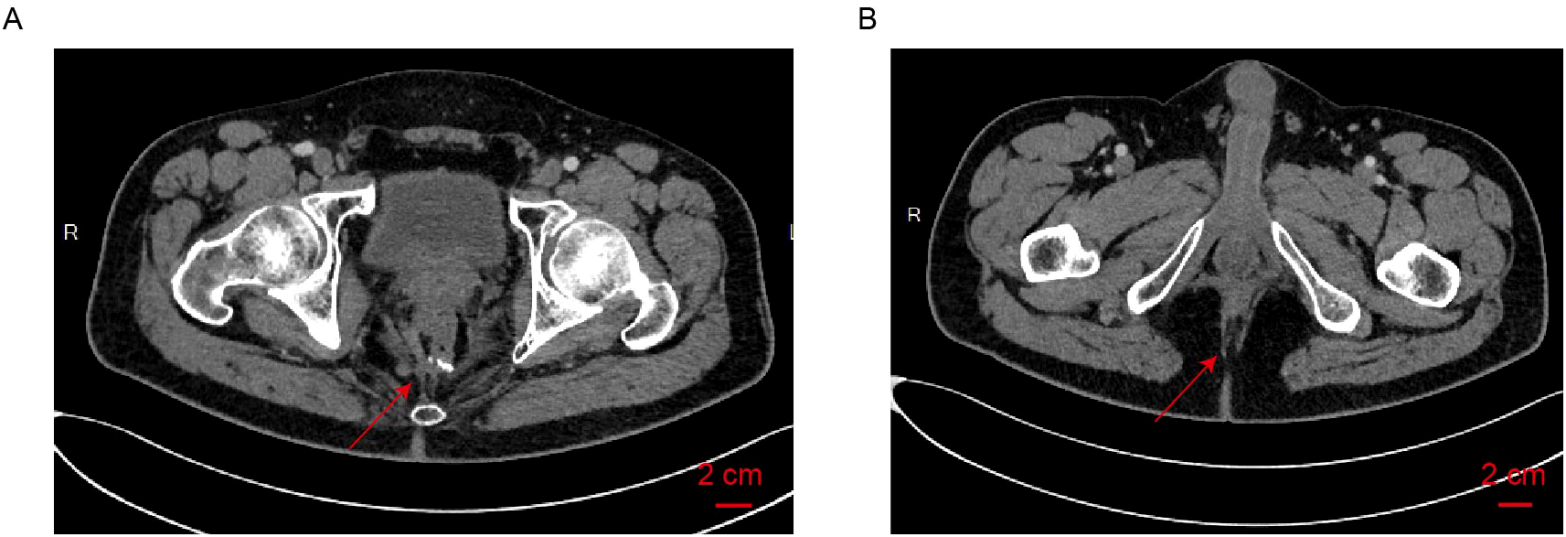
No evidence of tumor recurrence or metastasis was observed on the most recent CT examination (2025-09-24). **(A)**: Axial CT of the rectal anastomosis. The arrow indicates the rectal anastomosis with no evidence of local recurrence. **(B)**: Axial CT of the anus. The arrow indicates anus with no evidence of local recurrence.

The patient underwent regular postoperative surveillance. Throughout follow-up, he reported high satisfaction with preserved sphincter function and maintained a favorable quality of life. At the most recent follow-up in September 2025, CT and endoscopic evaluations showed no evidence of tumor recurrence or distant metastasis (**Table 1**; **Figure 2**). Serum tumor markers, including carcinoembryonic antigen (CEA) and carbohydrate antigen 19-9 (CA19-9), were routinely monitored and remained within normal reference ranges throughout the entire clinical course (January 2022–September 2025). The patient continues to be disease-free at the latest follow-up.

## Discussion

This case report demonstrates the successful treatment of metachronous LARC using a combination of intratumoral H101 and TNT. The patient achieved a cCR and pCR response and retained anal function. To our knowledge, this is the first case report of H101 intratumoral therapy combined with TNT in the treatment of rectal cancer.

TNT is increasingly recognized as a game-changer in the treatment of LARC. TNT delivers both systemic and localized therapy prior to surgical intervention, aiming to maximize tumor regression before resection (8). Studies have demonstrated that TNT significantly improves pCR rates, achieving up to 30–40% compared to conventional neoadjuvant chemoradiotherapy (6). The RAPIDO trial (2021) found that short-course radiotherapy followed by chemotherapy before total mesorectal excision resulted in a higher rate of distant metastasis-free survival and lower treatment-related toxicity (3). Similarly, the UNICANCER-PRODIGE 23 trial (2021) confirmed the superiority of TNT over conventional treatments in terms of long-term overall survival, disease-free survival and metastasis-free survival (17). These findings emphasize the transformative potential of TNT in achieving better oncological and functional outcomes. Moreover, it allows for better sphincter preservation, a critical factor in patients where rectal surgery might lead to permanent colostomy (18). However, despite its advantages, TNT is not without challenges. Some patients experience increased toxicity, leading to concerns about treatment adherence (19). Additionally, not all tumors respond optimally, necessitating novel therapeutic strategies such as immunotherapy (20) and OVT (15).

Oncolytic viruses (OVs) are a class of genetically engineered viruses that can selectively infect tumor cells. OVT provides novel and promising therapeutic options for patients with cancers resistant to traditional therapies. OVT destroys tumor tissues via multiple mechanisms: direct oncolysis of tumor cells, disrupting tumor vasculature and activating anti-tumor immunity (10). Unlike traditional cytotoxic treatments, OVT not only induce direct tumor cell death but also release of tumor-associated antigens. In addition, oncolysis results in the release of damage-associated molecular patterns, such as calreticulin, ATP, and HMGB1, which recruit cytotoxic T lymphocytes and dendritic cells to the tumor microenvironment (21). This promotes the activation of antigen-presenting cells, triggering an immune response that can eliminate residual tumor cells and prevent metastasis (22). The efficacy of OVT as a single agent has not yet been optimized for maximum anti-tumor effectiveness. However, OVT has emerged as a promising strategy for augmenting the efficacy of standard cancer treatments, including chemotherapy, radiotherapy, and immunotherapy (23).

Currently, several studies are exploring the safety and efficacy of OVT in CRC. A Phase 1 clinical trial (NCT03916510) is investigating the intravenous injection of the novel oncolytic virus Enadenotucirev in combination with chemoradiotherapy for LARC (24). In the high-dose group of Enadenotucirev, three out of six participants exhibited no macroscopic evidence of residual tumor, suggesting a potential therapeutic benefit. However, two of six patients experienced serious adverse events. Recent research interest has increasingly shifted toward combining OVT with immunotherapeutic strategies (25). One such study (NCT01394939) is evaluating the oncolytic virus JX-594 (Pexa-Vec) as monotherapy or in combination with irinotecan for CRC patients who are resistant or intolerant to standard treatments. In this non-randomized study, no objective response (OR) was observed in the monotherapy group (0/25), whereas one patient in the combination group (1/17) achieved a partial response. However, the intravenous administration of JX-594 at a dose of 1 × 10^9^ PFU on days 1, 8, 15, 22, and 29 was associated with a high rate of serious adverse events (40%, 10/25). In contrast, a Phase I/II study (NCT03206073) investigating Pexa-Vec in combination with immune checkpoint inhibitors—durvalumab (anti–PD-L1) and tremelimumab (anti–CTLA-4)—in patients with refractory metastatic CRC demonstrated that this combination (3 × 10^8^ PFU, same administration schedule) was safe and well tolerated, with no unexpected toxicities reported (26). In addition to these ongoing trials, further investigations into next-generation oncolytic viruses in combination with immunotherapy and targeted therapies are also underway, including NCT06283303 and NCT05733611.

From these studies, it is evident that the use of oncolytic viruses in colorectal cancer treatment faces significant challenges in terms of efficacy, safety, and targeting. First, the monotherapy efficacy of oncolytic viruses remains limited. Second, intravenous administration of OVs may pose safety risks, particularly in immunocompromised patients. There are still uncertainties regarding the optimal dosing and injection methods for OVs. Third, the overall low virus delivery efficiency in systemic administration is a concern, as host antiviral immune responses limit therapeutic efficacy. Therefore, further research is essential to explore different injection methods, doses, and combination therapies to improve the efficacy and safety of oncolytic virotherapy in colorectal cancer treatment.

H101 is a genetically modified, conditionally replicating oncolytic adenovirus. It preferentially replicates in tumor cells with defective p53 signaling. In contrast, normal cells with intact p53 function can suppress viral replication, resulting in selective tumor cell cytotoxicity (14). Previous clinical studies have shown that intratumoral oncolytic adenovirus H101 can induce tumor regression and improve disease control, either as monotherapy or in combination with systemic therapy or concurrent chemoradiotherapy, with an acceptable safety profile (11, 12). H101 is currently approved by the China National Medical Products Administration (NMPA) for patients with advanced nasopharyngeal carcinoma who are insensitive to radiotherapy or cannot undergo surgery (27). In addition, clinical trials are currently underway in multiple tumors, including cervical cancer (11), hepatocellular carcinoma (25), melanoma (ChiCTR2000037761), and CRC (28).

It is still unknown whether OVT combined with TNT has a synergistic effect in treating tumors. Several hypotheses for the mechanism of synergy between OVT and chemotherapy can be formulated. Chemotherapeutic drugs induce DNA damage and inhibit antiviral mechanisms, making tumor cells more susceptible to viral infection and replication (29). Radiotherapy induces double-stranded DNA breaks and promotes viral replication. In addition, radiotherapy can also induce increased tumor vascular permeability, promoting targeted delivery of viruses (30).

In a recent study, researchers conducted a retrospective analysis of 20 patients with locally advanced cervical cancer who received intratumoral injections of the oncolytic virus H101 in combination with concurrent chemoradiotherapy. The results demonstrated that H101 could effectively promote regression of the primary tumor while maintaining an acceptable safety profile. The most commonly observed H101-related adverse event was fever, reported in 91.3% of patients, which was generally manageable (11).

These findings support the potential of H101 as an effective adjunct to standard treatment protocols, and provide a rationale for extending its application to other solid tumors such as rectal cancer. In our case, the combination of TNT and intratumoral H101 injection led to substantial tumor regression while successfully preserving anal sphincter function. The patient expressed high satisfaction with postoperative anal function, and no evidence of local recurrence or distant metastasis was observed at the last follow-up.

This case presents several noteworthy features. First, the tumor was located in close proximity to the anal verge, facilitating direct and accurate intratumoral delivery of the oncolytic virus. Second, genomic profiling of the tumor revealed a TP53 mutation, which may be relevant to the virus’s selective replication and therapeutic efficacy, as TP53-deficient tumor cells are known to be more susceptible to oncolytic adenovirus-H101. These unique characteristics highlight the clinical feasibility and biological rationale for combining H101 with TNT in selected patients, and underscore the need for prospective clinical trials.

This study has several limitations. First, this is a single-case report, and the findings may not be generalizable. Second, although the patient remains disease-free at the most recent follow-up, the overall follow-up duration is relatively limited, and long-term oncologic outcomes require further observation. Third, potential selection bias cannot be excluded, as treatment decisions were individualized based on clinical considerations. Finally, the absence of serial tissue and blood sampling precluded direct assessment of viral replication, intratumoral viral persistence, and immune microenvironment changes. Prospective studies with larger cohorts and systematic translational analyses are warranted to better define the contribution of oncolytic virotherapy within total neoadjuvant treatment strategies.

## Conclusion

The combination of recombinant human adenovirus type 5 (H101) and total neoadjuvant therapy shows great promise as a treatment strategy for locally advanced rectal cancer. This case highlights the potential of H101 to enhance chemotherapy and radiotherapy effects. Further research and clinical trials are needed to confirm the efficacy and safety of this combination approach, as well as to explore its potential for other malignancies.

## Data Availability

The datasets presented in this study can be found in online repositories. The names of the repository/repositories and accession number(s) can be found in the article/supplementary material.
